# Formulation and Evaluation of Fluconazole Containing Sodium Alginate/Methylcellulose-Based Buccal Films for Potential Treatment of Oral Candidiasis

**DOI:** 10.3390/pharmaceutics18060748

**Published:** 2026-06-18

**Authors:** Adekunle Oduneye Odularu, Anuoluwapo Temitope Adesegun, Chukwuemeka Paul Azubuike, Oluwadamilola Miriam Kolawole

**Affiliations:** 1 Department of Pharmaceutics and Pharmaceutical Technology, University of Lagos, Lagos +23401, Nigeria; odular1@yahoo.com (A.O.O.); desegunanu@gmail.com (A.T.A.); cazubuike@unilag.edu.ng (C.P.A.); 2 School of Pharmacy, De Montfort University, The Gateway, Leicester LE1 9BH, UK

**Keywords:** sodium alginate, methylcellulose, fluconazole, buccal films, oral candidiasis, physicomechanical, mucoadhesion, antifungal, drug content, drug release

## Abstract

**Background/Objectives**: Oral candidiasis is an infection of the oral cavity caused by *Candida albicans*. Mucoadhesive buccal films could adhere to the buccal mucosa for prolonged periods, improving the therapeutic outcomes of patients with oral candidiasis. This study aimed to develop and evaluate the properties of fluconazole containing sodium alginate/methylcellulose-based buccal films for potential treatment of oral candidiasis. **Methods**: Drug-polymer compatibility was investigated using FT-IR spectrophotometry. Three optimised fluconazole films (F1 to F3) containing 1–1.6% sodium alginate and methylcellulose (1.6%) were formulated using the solvent-casting method. Their physicomechanical properties were characterised using standard protocols. Drug content and in vitro drug release profiles were evaluated using UV-visible spectroscopy; in vitro/ex vivo mucoadhesion studies were conducted using the shaking water bath technique, and their antifungal activity against *Candida albicans* was evaluated using the agar ditch method. **Results**: FT-IR data analysis revealed that sodium alginate, methylcellulose and fluconazole were compatible in the films. The films were off-white, smooth, peelable, thin, with satisfactory pH values, folding endurance, drug content, excellent zones of inhibition against *Candida albicans* (40 mm), controlled drug release profile (3.6–4.1 mg/cm^2^ after 6 h), and they displayed Korsmeyer–Peppas drug release kinetics. Film F3 containing 1.6% sodium alginate and 1.6% of methylcellulose exhibited superior swelling index (70 ± 1%), tensile strength (0.68 ± 0.04 MPa) and in vitro/ex vivo mucoadhesion time (5.5 ± 0.3 h; 2.3 ± 0.3 h) relative to other studied films. **Conclusions**: The sodium alginate content of the films influenced their tensile and mucoadhesive properties. Film F3 was the most promising formulation for potential treatment of oral candidiasis.

## 1. Introduction

Candidiasis (oral thrush) is primarily caused by a fungus, *Candida albicans*, the most prevalent species found in the human oral cavity [1]. Patients with oral candidiasis present with absence of taste or an unpleasant taste in the mouth; inflamed mouth and throat; and painful, burning sensation in the mouth [2]. The disease is rarely prevalent amongst healthy adults of the general population [1]. Predisposing factors to developing oral candidiasis include infants, smoking, diabetes mellitus, cancer, and immunocompromised conditions such as HIV infection [1,3]. Approximately 95% of individuals with HIV experience oral candidiasis during their illness, and it is often a prognostic indicator of AIDS [4,5]. Given the high incidence and associated morbidity of oral candidiasis, effective treatment of the disease is crucial, particularly in populations with weakened immune systems.

Fluconazole (2,4-difluoro-α 1,3-bis (1H-1,2,4-triazol-1-yl) propan-2-ol), a synthetic triazole fungicidal drug, is one of the approved drugs for treating moderate to severe cases of oral candidiasis [6]. Fluconazole exerts its fungicidal activity by inhibiting the enzyme, lanosterol 14-α-demethylase, resulting in improved permeability of the fungal cell membrane to fluconazole, and resulting in fungal cell death [7].

The clinical effectiveness of conventional fluconazole formulations such as tablets, suspensions, and infusions is limited by the extensive systemic distribution of fluconazole, resulting in an increased risk of systemic toxicity. In addition, oral formulations are degraded by stomach acid and liver enzymes, leading to subtherapeutic concentrations of fluconazole within the oral tissues [8]. Though moderate to severe cases of oral candidiasis have been effectively treated by administering 100–200 mg of fluconazole orally once daily for 7 to 14 days, long duration of treatment may result in poor patient compliance [6]. Moreover, systemic therapies are associated with poor patient acceptance due to its invasiveness. Therefore, buccally delivered drug formulations could be developed to facilitate localised drug delivery, minimise systemic exposure, reduce the potential for adverse effects, and promote effective treatment of oral candidiasis.

Buccal films comprise film-forming agents, mucoadhesive polymers, plasticisers, and polymers that can facilitate controlled drug release [9]. Glycerol and propylene glycol are the most commonly used plasticising agents that improve the flexibility of film formulations [9]. Examples of mucoadhesive polymers employed to prepare buccal film formulations include chitosan, sodium alginate and hydroxypropylmethylcellulose [10,11,12]. Mucoadhesive films attach to the oral mucosa, facilitating sustained release of the loaded drug for prolonged periods, enhancing treatment efficacy and patient compliance due to reduced frequency of dosing.

Over the last two decades, mucoadhesive buccal films have been well-researched due to flexibility, convenience of application, their capability to avoid gastric acidic and hepatic enzymatic drug degradation as well as their ability to deliver therapeutic agents for local or systemic therapeutic effects [13]. In addition, buccal films can be customised to accommodate required amount of the therapeutic agents, enabling personalised medicines [9].

Different buccal products were investigated for local and systemic drug delivery. For instance, fluticasone-, felodipine- and haloperidol-containing buccal films were used systemically to control asthma, blood pressure, and schizophrenia, respectively [14,15,16] while cetylpyridinium-, chloride-, and doxycycline hyclate-containing buccal films have been studied for localised drug effect to treat bacterial infections and periodontitis, respectively [10,17].

Various buccal products that exert systemic therapeutic effects, such as buprenorphine (Belbuca) and fentanyl (Onsolis), have been commercialised [18,19]. To our knowledge, there are no commercialised drug-containing buccal film formulations for the treatment of localised buccal diseased conditions, including oral candidiasis [20]. This gap highlights the need for further research into buccal film formulations that can deliver antifungal agents, such as fluconazole, in a controlled manner, for the effective treatment of oral candidiasis.

Fluconazole is suited for buccal drug delivery because the drug can effectively permeate the oral mucosa due to its satisfactory physicochemical properties (molecular weight: 306.3 Da; log P: 0.4) [21]. The development of mucoadhesive fluconazole-containing buccal film could facilitate a reduction in the therapeutic dose of fluconazole from 200 mg to 5 mg and minimise side effects associated with disease treatment via the oral and systemic route of administration due to its localised drug delivery capability.

Sodium alginate is a linear, hydrophilic, anionic polymer naturally found in marine brown seaweed, which is composed of (1,4)-linked *b*-D-mannuronic acid units and *a*-L-guluronic acid units [22]. It has intrinsic mucoadhesive property due to its carboxylic groups that interact with the hydroxyl groups of mucin glycoproteins [23]. Methylcellulose is a semi-synthetic, hydrophilic, non-ionic polymer that has mucus-penetrating potential, and it can facilitate controlled drug release [24]. In addition, both sodium alginate and methylcellulose have film-forming properties [25]. Moreover, the Food and Drug Administration, USA acknowledges the safety of these polymers [26]. Therefore, they have been selected in the current study to formulate mucoadhesive fluconazole-containing buccal films for the potential treatment of oral candidiasis.

Patel and coworkers reported that optimised sodium alginate (SA)/polyvinylalcohol (PVA)-based fluconazole films containing 1% SA and 1% PVA exhibited satisfactory physicomechanical properties, ex vivo rat skin residence time of 8 h, and controlled drug release profile over 8 h [27]. However, the anti-fungal activity of the optimised fluconazole-containing buccal film was not investigated. Therefore, the clinical translation of the film formulation could not be verified.

Dinte et al. (2023) revealed that doxycycline hyclate-containing hydroxypropyl methylcellulose (HPMC) E3/HPMC K4/Carbopol 940 (1:0.05:0.35) was the best film for the possible treatment of periodontitis in terms of its physicomechanical, mucoadhesive, controlled drug release profile, non-irritancy to the buccal tissues and efficacy against experimentally induced periodontitis in rat models [12]. Nevertheless, their newly developed buccal films are not intended to treat oral candidiasis.

Posaconazole is typically used to treat oral candidiasis when the disease is resistant to itraconazole and fluconazole therapy. Recently, Szekalska et al. reported that optimised posaconazole-containing calcium carbonate-crosslinked alginate/pectin-based buccal films prepared using 1% of sodium alginate and 1% pectin exhibited improved mechanical properties and facilitated controlled drug release compared to the non-crosslinked films. However, the calcium carbonate crosslinking process compromised the film formulation’s mucoadhesiveness and antifungal activity against *Candida albicans* [11]. Therefore, the novel posaconazole-containing buccal film may result in therapeutic failure in the clinics due to the limited interaction between the posaconazole-containing films and the diseased buccal mucosal tissues. Moreover, the film formulation was not suited for the first line treatment of oral candidiasis.

To our knowledge, buccal films have never been commercialised for the treatment of oral candidiasis. Sodium alginate will provide mucoadhesive effect to the newly developed film while methylcellulose will facilitate controlled fluconazole release. The combination of sodium alginate, methylcellulose, and appropriate plasticiser could generate an aesthetically appealing, quality and efficacious buccally delivered dosage form. The novel buccal film could facilitate fluconazole dosage reduction from 150 mg (oral administration) to 5 mg (buccal delivery). Also, the influence of increasing amounts of sodium alginate with a constant concentration of methylcellulose, on the properties of fluconazole films will be investigated. This study aimed to formulate fluconazole-containing sodium alginate/methylcellulose-based buccal films and evaluate their physicomechanical, mucoadhesive, drug release, stability, and antifungal properties for potential treatment of oral candidiasis.

## 2. Materials and Methods

### 2.1. Materials

Sodium Alginate (Merck, Dorset, UK), methylcellulose, low substitution (FengChen, Ltd., Dongying, China); propylene glycol (Dongying Runze New Materials Ltd., Dongying, China), fluconazole (Macklin, Shanghai, China), phosphate buffer (pH 6.8), dialysis membrane (MWCO12–14 kDa) (Medicell Membranes Ltd., London, UK); and cellulose membrane (0.45 μm; Merck, Dorset, UK), Sabouraud dextrose agar, and nutrient agar were used. Deionised water was used for all experiments. All other reagents were of analytical grade and used as supplied.

### 2.2. Ethical Approval of Studies Involving Porcine Buccal Mucosal Tissues

The ex vivo porcine buccal mucoadhesiveness of the novel fluconazole films was evaluated after obtaining research ethical approval (CMUL/ACUREC/08/24/1696), dated 17 November 2024. The porcine buccal mucosal tissues were excised from freshly slaughtered pigs intended for food and transported over ice-cold water to the laboratory and used within 24 h of procurement. The location of the abattoir is Lagos, Nigeria. The work was carried out according to the regulations of the College of Medicine, University of Lagos Animal Care and Use Research Ethics Committee, Lagos, Nigeria.

### 2.3. Methods

#### 2.3.1. Fluconazole/Polymer Compatibility Studies—Attenuated Transmittance Reflectance–Fourier Transform Infrared (ATR-FTIR) Spectroscopy

Fluconazole, sodium alginate and methylcellulose (solids), alone and in combination with other ingredients (1:1), and used to formulate fluconazole film, were scanned using the ATR-FTIR spectrometer (CARY 630; Agilent Technologies, Santa Clara, CA, USA), from 4000 to 650 cm^−1^, at a resolution of 8 cm^−1^, and 32 scans were conducted for each tested sample to generate the FT-IR data. This procedure was also conducted on optimised film formulations to confirm that fluconazole remained compatible with the polymers after buccal film development.

#### 2.3.2. Preparation of Fluconazole-Containing Buccal Films

Sodium alginate/methylcellulose-based fluconazole-containing buccal films were prepared using solvent casting technique [24]. Table 1 details the compositions of the film formulations. First, sodium alginate and methylcellulose were dispersed in predetermined volume of water and maintained under magnetic stirring for 1 h. Then, the calculated amount of fluconazole and plasticiser (glycerol or propylene glycol) was added to the film-forming solution, and the film forming solution was stirred for an additional 1 h. Afterward, 45 mL of each drug-containing film-forming solution was transferred into 90 mm glass Petri dishes and dried in an oven (Morex Medical, London, UK) at 60 °C for 20–24 h. The dried fluconazole film formulations were secured in baking paper/aluminium foils and maintained in a desiccator at room temperature.

### 2.4. Physicomechanical Evaluation

#### 2.4.1. Organoleptic Studies

The colour, odour, peelability, texture and homogeneity of the fluconazole-containing buccal films were assessed by sensory examination. The colour and homogeneity of the films were determined visually while the odour of the samples was determined by nasal perception. Three samples were tested per film formulation (n = 3).

#### 2.4.2. Weight Uniformity Evaluation

The extent of weight uniformity of the films was evaluated by weighing five films (1 cm × 1 cm) using an analytical balance (YIWU Dawood Import & Export Co., Ltd., Dongyang, China) and the mean weight of each film was determined and expressed as mean ± SD (n = 5).

#### 2.4.3. Film Thickness Evaluation

The thickness values of the optimised films (1 cm^2^) were evaluated using a micrometre screw gauge (Japan Scientific Co., Ltd., Tokyo, Japan), and the mean film thickness values were recorded (n = 5) [24].

#### 2.4.4. Surface pH

The surface pH values of the optimised fluconazole films (2 cm × 2 cm) were evaluated using a previously reported method, with simulated salivary fluid used for experimentation [24]. The fluconazole-containing buccal films were placed in various Petri dishes containing 2 mL of simulated salivary fluid (pH 6.8), and the films were allowed to swell for 15 min. Then, the surface pH values of the hydrated film formulation were determined using a digital pH metre (PH-3012B, Shenzhen Tomtop Technology Co., Ltd., Shenzhen, China). The pH evaluation test was carried out in triplicates (n = 3).

#### 2.4.5. Folding Endurance

The folding endurance values of the optimised buccal films were evaluated by repeatedly folding the films (1 × 2 cm^2^) at the same axis; and counting the number of folds, the film tolerated before puncture [24]. The experiment was carried out in triplicates (n = 3).

#### 2.4.6. Tensile Test

The tensile potential of the optimised fluconazole-containing buccal film formulations were evaluated using a published method [24]. The tensile properties of the polymeric films (1 cm × 3 cm rectangular sections) were investigated using the Universal Testing Machine (Instron-series 3369, Instron Corporation, Norwood, MA, USA), fitted with a 50 kN load cell. The sections of the film formulations were mounted between two clamps and stretched at a rate of 50 mm/min with an applied load and gauge length of 50 N and 5 cm, respectively. The experiment was conducted at 20 °C and relative humidity of 60%. The study was carried out in triplicates (n = 3).

The tensile strength of the film is the highest amount of tensile stress that the film formulation can bear before puncture while the percent elongation at break- (EOB) or breaking-strain value of the film is shown in Equation (1).

(1) $$Elongation\;at\;Break\left(\%\right)=\frac{Change\;in\;film\;length}{initial\;film\;length}\times 100$$

### 2.5. Swelling Index—Percent Hydration

#### 2.5.1. Preparation of Simulated Salivary Fluid

Phosphate-buffered solution (pH 6.8, 0.2 M), which served as the simulated salivary fluid, contained 13.872 g of potassium dihydrogen phosphate and 35.084 g of disodium hydrogen phosphate dissolved in deionized water (200 mL) and stirred for 1 h. The pH of the buffer solution was adjusted to pH 6.8 before making up the buffer solution to the required volume (250 mL).

#### 2.5.2. Evaluation of Swelling Index

The swelling indexes of the optimised film formulations were evaluated as a function of simulated salivary fluid hydration of the films. Briefly, each film (1 cm^2^) was weighed (W1) and dipped in simulated saliva (PBS pH 6.8; 10 mL) maintained at 37 ± 1 °C. At predetermined time intervals, the film was removed from the simulated saliva (5, 10, 15, 20, 25, 30, and 35 min), and the film was wiped and reweighed (W2) [24]. The experiment was carried out in triplicates (n = 3).

The percentage film hydration was calculated using Equation (2).

(2) $$Film\;hydration\left(\%\right)=1-\frac{W1}{W2}\times 100$$

#### 2.5.3. Disintegration Time Evaluation

The swelling of the fluconazole films was followed by disintegration. Therefore, the time (min) required for the film to stop swelling and fully disintegrate was recorded as the “disintegration time”. The experiment was done in triplicates (n = 3).

### 2.6. In Vitro Adhesion Studies

The in vitro adhesion time values of the fluconazole film formulations were evaluated using a published technique [24]. The pre-hydrated cellulose membrane (2 × 3 cm^2^) was fixed to the surface of a glass slide (2.5 × 7.5 cm) using an adhesive. The surface of a buccal film (1 × 1 cm^2^) was wetted using phosphate-buffered saline (PBS; pH 6.8). The test film formulation was secured on the surface of the cellulose membrane (simulant buccal membrane). Then, PBS (100 mL) was poured into the beaker and the glass slide securing the membrane and film formulation was placed into the beaker at an angle of 45° such that the membrane and film were completely immersed in the simulated saliva. Afterward, the beaker was secured in a shaker water bath (SHA-C; Etrack Scientific Instruments, London, UK) maintained at 37 °C and agitated at 75 rpm to mimic buccal physiological conditions. The time required for detachment of the fluconazole film from the simulant buccal membrane was noted as the in vitro adhesion time (n = 3).

### 2.7. Ex Vivo Porcine Buccal Mucoadhesion Studies

The ex vivo porcine buccal mucoadhesion values of the fluconazole films were determined using the technique described in Section 2.6, with the simulant buccal (cellulose) membrane replaced with porcine buccal mucosal tissues. Briefly, buccal mucosal tissues were obtained from freshly slaughtered pigs collected from the local abattoir, and the animal tissues were transported over ice-cold water (pH 7.0 ± 0.2) to the laboratory. Excess connective tissues were trimmed using a scalpel blade and the buccal mucosal surfaces were protected from damage during tissue preparation. Then, the buccal mucosal tissues (2 cm × 3 cm) were rinsed using phosphate buffer (pH 6.8) prior to experimentation and stuck to a glass slide (2.5 × 7.5 cm) using an adhesive. The fluconazole film (1 cm × 1 cm) was attached to the glass slide securing the buccal tissues (2 cm × 2 cm). Then the glass slide was secured in a beaker containing phosphate buffer (100 mL; pH 6.8) at 45°, and the beaker was maintained in a water bath agitated at 75 rpm to mimic buccal environment. The ex vivo buccal mucoadhesion time, which is the time taken for the studied films to detach from the buccal mucosal tissues, was noted (n = 3).

### 2.8. Mean Drug Content Evaluation

Buccal film formulation (1 × 1 cm^2^) that should contain 5 mg of fluconazole was dispersed in methanol (80 mL) and stirred magnetically for 30 min. Then, the drug solution was filtered and made up to 100 mL drug solution in a volumetric flask. Afterward, the drug solution was agitated in an ultrasonic bath. In addition, pure fluconazole (5 mg) was solubilised in methanol, filtered and made up to 100 mL in a volumetric flask using methanol. The samples were analysed using a UV-visible spectrophotometer (UV-6300PC, VWR Instruments, Mississauga, ON, Canada) at the wavelength of maximum fluconazole absorption (208 nm). The experiment was carried out in triplicates (n = 3).

The percent fluconazole content (Equation (3)) was determined as follows:

(3) $$\%\;Drug\;Content=Absorbance\;of\;\frac{drug\;loaded\;film}{pure\;drug}\times 100$$

### 2.9. In Vitro Fluconazole Release Studies

Simulated salivary fluid was prepared (described in Section 2.5) and mixed with methanol (1:1) and the pH of the drug release medium was adjusted to pH 6.8 using 0.1 M hydrochloric acid solution. The drug release experiment was carried out using the Franz diffusion cells. A pre-hydrated cellulose membrane (Millipore, Burlington, MA, USA, 0.45 μm) was secured between the donor and acceptor medium of the Franz cell. Then, simulated salivary fluid/methanol (1:1; 20 mL, pH 6.8) was put into the acceptor compartment of the Franz cell, and the Franz cell was placed on a magnetic stirrer maintained at 37 °C, which was continuously stirred throughout the experiment. The Franz cell securing the membrane and fluconazole film was equilibrated for 30 min at 37 °C before the hydrated fluconazole film (1 × 1 cm) was placed in the donor compartment. Aliquots (2 mL) were withdrawn from the acceptor compartment at predetermined periods (0.5, 1, 2, 4, and 6 h) and replaced with an equal amount of fresh prewarmed simulated salivary fluid to maintain sink conditions. The drug content of aliquot samples was analysed at the wavelength of maximum absorption for fluconazole (261 nm) using a UV-visible spectrophotometer (UV-6300 PC; VWR Instruments, Mississauga, ON, Canada) and UV method that is validated (Table S1). The experiment was carried out in triplicates (n = 3). Standard solutions of fluconazole in a simulated salivary fluid/methanol mixture, pH 6.8 (1:1) (5 μg/mL to 100 μg/mL), were used to construct a standard calibration curve that quantified the amount of fluconazole released from the buccal films (n = 3).

The fluconazole release data was fitted into various drug release kinetic models (zero-order, first-order, Higuchi and Korsemeyer–Peppas model equation) to determine the drug release kinetics of the fluconazole film formulations [24].

### 2.10. Antifungal Activity of Fluconazole Films Against C. albicans

The antifungal profiles of fluconazole-containing buccal films against *Candida albicans* were studied using a Sabouraud agar ditch method [24]. Briefly, each fluconazole film (1 × 1 cm^2^) was placed into a ditch created at the centre of the agar plate and freshly prepared calibrated *Candida* culture loop was applied at a right angle from the ditch to the edge of the agar-containing Petri dish. Then, the plate was maintained at 25 °C for 72 h, with fluconazole solution serving as the positive control. Afterward, zones of fungal inhibition were recorded for the optimised tested drug-containing samples. The experiment was carried out in triplicates (n = 3).

### 2.11. Real-Time Stability Studies

The real-time stability profiles of the optimised fluconazole-containing buccal film formulations were evaluated in compliance with the ICH guidelines for intermediate stability evaluation [28]. The buccal films were wrapped in baking paper/aluminium foil and stored at 30 ± 2 °C/65 ± 5% RH for six months. The fluconazole films were observed monthly for alterations in their organoleptic attributes and folding endurances and observations were noted (n = 3).

### 2.12. Statistical Analysis

All experimental data were presented as mean ± standard deviation. Statistical analysis of research data was conducted using One-Way ANOVA/Bonferroni post hoc test, with *p* < 0.05 signifying significant statistical differences between datasets.

## 3. Results

### 3.1. Drug–Polymer Compatibility Profile

**Figure 1:**
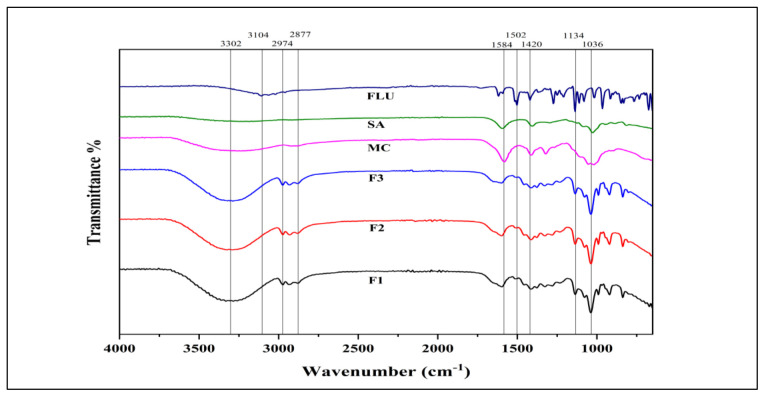
Overlapped spectra of fluconazole, sodium alginate, methylcellulose, and fluconazole films F1 to F3; F1 = SA1%/MC1.6%, F2 = SA1.3%/MC1.6%, F3 = SA1.6%/MC1.6%.

The FTIR spectra of fluconazole, methylcellulose, sodium alginate, and the optimised fluconazole film formulations are shown in Figure 1. The functional groups present in sodium alginate and methylcellulose were similar: Absorption bands at 1017–1021 cm^−1^ depicting C-O-C glycosidic bonds and those at 1401–1408 cm^−1^ indicated symmetric stretching of COO groups in sodium alginate. Fluconazole, sodium alginate and methylcellulose had peaks appearing at 1584–1587 cm^−1^, suggesting C-C stretch in ring and asymmetric stretching of COO groups, while absorption bands at 2843–3660 cm^−1^ represented their C-H and O-H stretch. In addition, fluconazole had a distinctive C-N stretch at 1140–1274 cm^−1^.

### 3.2. Organoleptic Properties

Fluconazole-containing sodium alginate/methylcellulose-based buccal films were successfully formulated using propylene glycol as the plasticiser. The percentage of components (Table 1) referred to 100 mL of film-forming solution and not the final weight of the film, while fluconazole dose is related to the drug loaded per square centimetre of the film.

**Figure 2:**
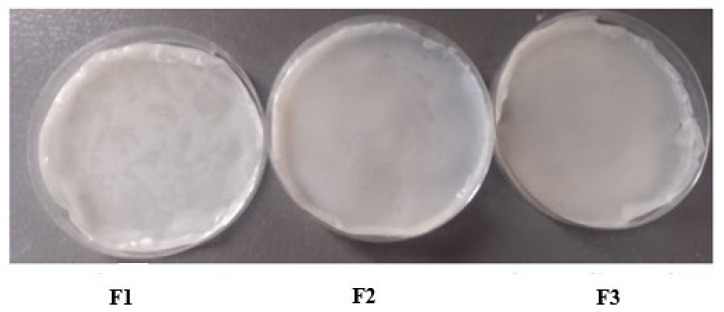
Image of fluconazole films with differing sodium alginate content; F1: Fluconazole/1% SA/1.6% MC; F2: Fluconazole/1.3% SA/1.6% MC; F3: Fluconazole/1.6% SA/1.6% MC.

Figure 2 showed the three optimised fluconazole-containing films with varying concentrations of sodium alginate while maintaining a constant concentration of methylcellulose (1.6% w/w) and plasticiser (10%) across all samples. The films were off-white, odourless, peelable, homogenous, with smooth texture.

### 3.3. Physicomechanical Properties of Fluconazole Films

The mean weights of the fluconazole films (1 cm × 1 cm) ranged from 0.1 g to 0.12 g (Table 2).

Also, the thickness, surface pH, folding endurance values, as well as swelling indexes and disintegration time values of the optimised fluconazole films (F1, F2, and F3) are presented in Table 2.

### 3.4. Tensile Properties of Buccal Films

**Figure 3:**
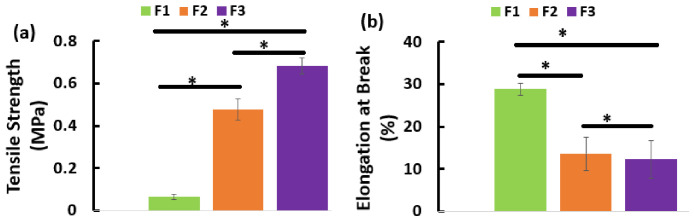
(**a**) Tensile strength and (**b**) elongation at break profiles of fluconazole buccal films; data presented as mean ± SD; n = 3; “*“ indicate significant statistical differences between datasets; key: F1: Fluconazole/1% SA/1.6% MC; F2: Fluconazole/1.3% SA/1.6% MC; F3: Fluconazole/1.6% SA/1.6% MC.

The tensile strength and breaking strain values of the optimised films are provided in Figure 3a,b.

### 3.5. In Vitro Adhesion and Ex Vivo Mucoadhesion Time Profiles

**Figure 4:**
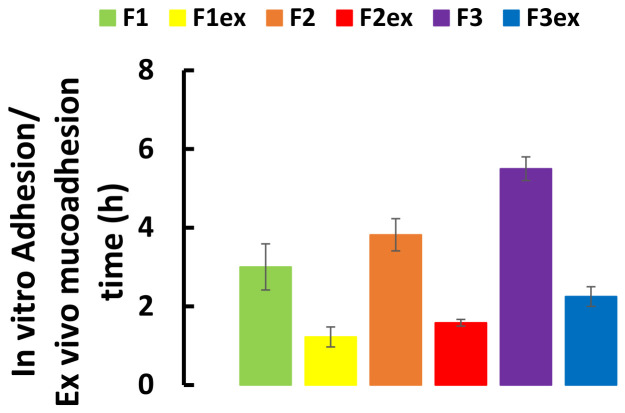
In vitro adhesion and ex vivo porcine buccal mucoadhesion time profiles of fluconazole films at 37 °C; key: F1: Fluconazole/1% SA/1.6% MC; F2: Fluconazole/1.3% SA/1.6% MC; F3: Fluconazole/1.6% SA/1.6% MC (in vitro adhesion time values); F1ex, F2ex and F3ex: Data from ex vivo mucoadhesion studies.

The mean time needed to detach the studied fluconazole buccal films from the simulated (cellulose membrane) or ex vivo buccal mucosa are the in vitro adhesion and ex vivo mucoadhesion time, respectively. The in vitro adhesion time increased in this order: film F1: 3.0 ± 0.6 h < F2: 3.8 ± 0.4 h < F3: 5.5 ± 0.3 h (Figure 4). The ease of detachment of the optimised fluconazole films from ex vivo buccal mucosal tissues exhibited a similar trend: Film F1: 1.2 ± 0.3 h < 1.6 ± 0.1 h < 2.3 ± 0.3 h.

### 3.6. Mean Drug Content Values

The mean drug content values of the optimised fluconazole films F1, F2 and F3 are 98.7 ± 0.2%, 99.7 ± 0.2%, and 113.8 ± 0.2%, respectively.

### 3.7. In Vitro Drug Release Profiles

#### 3.7.1. Standard Calibration Curve of Fluconazole in Simulated Saliva/Methanol (1:1; pH 6.8)

The fluconazole in simulated saliva/methanol mixture (1:1) calibration curve (Figure S1) revealed a regression coefficient (r^2^ value) of 0.9988 and the equation of the graph was y = 0.0132x.

#### 3.7.2. Fluconazole Flux Across Simulant Buccal Mucosa

**Figure 5:**
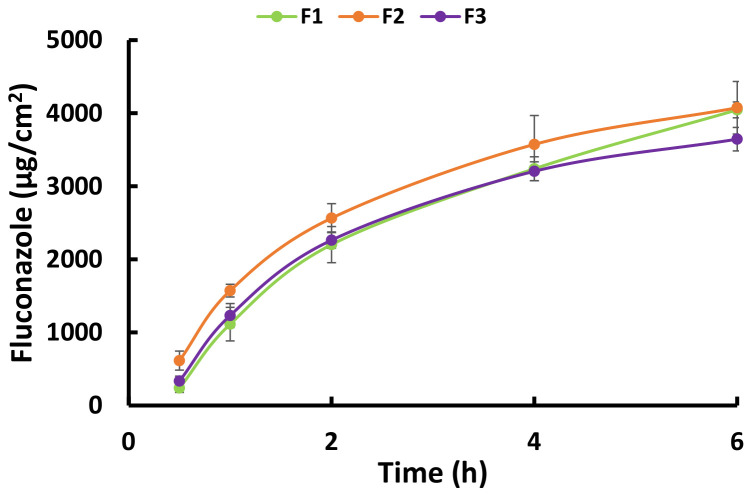
Fluconazole flux through simulant buccal mucosa over 6 h; release medium: pH 6.8; 37 °C; n = 3; key: F1: Fluconazole/1% SA/1.6% MC; F2: Fluconazole/1.3% SA/1.6% MC; F3: Fluconazole/1.6% SA/1.6% MC.

The values of fluconazole flux from fluconazole films F1 to F3 through the simulant buccal membranes are presented in Figure 5.

The result of fitting the fluconazole films’ drug release data to various drug release kinetic models (zero-order, first-order, Higuchi and Korsmeyer–Peppas models) are presented in Table 3.

### 3.8. Antifungal Profiles of Fluconazole Films Against Candida Albicans

The zones of fungal inhibition (ZOI) values for fluconazole solution and fluconazole film formulations (F1, F2, F3) against *Candida albicans* are shown in Table 4, and the images of the agar plate-revealing zones of *Candida* inhibition are shown in Supplementary Information, Figure S2.

### 3.9. Real-Time Stability Profiles

The fluconazole buccal films were stable at 30 ± 2 °C and a relative humidity of 65 ± 5% after 6 months. Films F1 to F3 demonstrated appealing colour (off-whitish), agreeable odour and folding endurance greater than 250, monthly, over 6 months.

## 4. Discussion

Oral candidiasis is an oral infection that is highly prevalent amongst immunocompromised patients such as individuals with HIV, cancer patients, people on prolonged intravenous steroid and antibiotic use, geriatrics and some paediatrics [6]. Fluconazole is one of the recommended therapeutic agents used to treat oral candidiasis [29]. Oral hygiene and topical fluconazole formulations are effective to treat mild cases of the disease while systemic fluconazole therapy is required for patients that are non-responsive to topical therapy or prone to developing systemic infections [6].

Conventional fluconazole formulations, such as oral capsules and tablets, induce systemic side effects due to the large volume of fluconazole distribution (0.55–0.65 kg/L) in systemic circulation [27]. Topical creams, lotions and sprays exhibit poor drug residence time, inaccurate drug dosing and variation in therapeutic performance. In addition, intravenous fluconazole therapies are invasive, which may limit patients’ compliance to dosage regimen, resulting in therapeutic failure [27].

The buccal route has been increasingly investigated in the last decade for local and systemic drug delivery due to its non-invasiveness, avoidance of gastric acidic and hepatic enzymatic drug degradation and facilitation of sustained drug release. Typically, contact between mucoadhesive buccal films and the buccal mucosa facilitate film swelling, disintegration, dissolution, drug release and permeation into underlying buccal tissues to facilitate effective disease treatment [27]. This study aimed to formulate mucoadhesive, thin, aesthethically appealing, and cost-effective buccal films using biocompatible, film-forming and mucoadhesive sodium alginate and methylcellulose. The optimised fluconazole films could enhance the therapeutic outcomes of patients with oral candidiasis. Also, they could serve as a substitute fluconazole formulation to commercialised oral fluconazole tablets and topical fluconazole creams, currently used in the clinics.

The FT-IR peaks recorded for fluconazole, sodium alginate and methylcellulose were evident in the three optimised fluconazole film formulations (Figure 1), and no new peaks were recorded. Nevertheless, there were shifts in the position at which the peaks appeared as well as varied peak intensities. This FT-IR result suggested that there was no remarkable chemical interaction between fluconazole and the polymers used to formulate the optimised buccal films. These findings are in good agreement with the FT-IR spectra of sodium alginate films reported by Moura-Alves et al., (2023) which revealed absorption peaks at 3251, 2933, 1598, 1407 and 1025 cm^−1^ [30]. The positions of these peaks are comparable with that of SA/MC-based films studied in this work.

Preformulation studies revealed that methylcellulose (1.8%) generated highly viscous polymeric dispersion that was difficult to incorporate with sodium alginate and fluconazole. On the other hand, formulation of fluconazole films using glycerol as the plasticiser yielded a sticky, non-peelable film product. This finding may be due to the hydrophilic nature of sodium alginate and methylcellulose requiring a hydrophobic plasticiser, such as propylene glycol, to generate a satisfactory film formulation. Therefore, the optimised fluconazole films were formulated using a constant amount of methylcellulose (1.6%) and varying amount of sodium alginate (mucoadhesive polymer). There was a progressive improvement in the homogeneity of the films as the concentration of sodium alginate in the fluconazole films increased from 1% to 1.6%. This could be due to the presence of more sodium alginate molecules facilitating hydrogen bonding with methylcellulose and promoting homogeneity of the film formulation.

The weight of pharmaceutical films should be light to facilitate patient’s comfort and acceptability. The low standard deviation values recorded during film weight measurement revealed that the film manufacturing process was reproducible, which is desirable to upscale film production. There were significant statistical differences in the weights of the films as their sodium alginate content increased from 1% to 1.6% (*p* < 0.05). The studied sodium alginate/methylcellulose-based fluconazole films were lighter than the cetylpyridinium chloride-loaded chitosan/polyvinylalcohol-based buccal films reported by Abouhussein and coworkers [10] (0.1–0.12 g versus 0.38–0.40 g). Therefore, the novel fluconazole film will be appealing to the patients due to their natural feel after buccal application, improving patient compliance to dosage regimen and therapeutic success.

Interestingly, all the optimised fluconazole films exhibited comparable thickness values (0.08–0.09 mm; *p* > 0.05). This finding revealed that an increase in the sodium alginate content of the films from 1% to 1. 6% did not have a remarkable impact on the film thickness. Cetylpyridinium chloride-containing buccal films displayed comparable thickness with the novel fluconazole films (0.11–0.12 mm versus 0.08–0.09 mm) [10]. On the other hand, the film thickness values of the new fluconazole films were greater than that of the myrtle extract-loaded gelatin/pectin/polyvinylpyrrolidone/methylcellulose-based oral films reported by Hashemi and coworkers (0.08–0.09 mm versus 0.03–0.04 mm) [31]. The thin myrtle extract-loaded oral films may be prone to puncture during handling, transportation and buccal administration, resulting in premature drug loss and short-lived therapeutic effect.

Previously reported hydroxypropyl cellulose/methylcellulose/ethylcellulose-based felodipine-containing buccal films, Eudragit RS 100/HPMC-based fluconazole oral strips, HPMCE3/HPMCK4/Carbopol 934-based doxycycline hyclate-based buccal films and Proloc/HPMC/Eudragit RS 100-based rizatriptan benzoate buccal films were thicker than the currently studied fluconazole films (0.12–0.17 mm versus 0.22–0.24 mm versus 0.35–0.45 mm versus 1.02–1.32 mm versus 0.08–0.09 mm) [12,13,15,32]. These findings revealed that the thickness values of buccal films are dependent on the type, number and concentration of polymeric excipients used to prepare the film formulations. Nevertheless, the newly developed fluconazole-containing buccal films (0.08–0.09 mm) would be appealing to the patient after buccal application due to their thin dimension. Also, their reduced thickness values could facilitate faster drug dissolution and permeation into underlying buccal mucosal tissues, resulting in the effective treatment of oral candidiasis.

The evaluation of the surface pH of buccal films is an indirect method of evaluating their buccal mucosal irritant potential. There is a strong need to develop buccal films which have surface pH comparable to the buccal pH (6.4) to avoid buccal mucosal irritation [33]. The acceptable pH for buccally delivered formulations could range from 5.5 to 7 to facilitate biocompatibility with the buccal mucosal membranes [17]. Interestingly, there was a statistically significant increase in the acidity of the films as the concentration of sodium alginate used to formulate the films increased from 1% to 1.3% (SA1%: pH 7.61 versus SA1.3%: pH 7.18) (*p* < 0.05). However, the pH values of films F2 and F3 containing 1.3% and 1.6% of sodium alginate, respectively, are statistically similar (pH 7.18 versus 7.13) (*p* > 0.05).

The novel fluconazole films F2 and F3 containing 1.3% to 1.6% sodium alginate displayed acceptable pH values for buccal drug administration (pH 7.1–7.2). The buccal irritant potential of the studied fluconazole films F1 to F3 (pH 7.13–7.61) may be minimal compared with that of cetylpyridinium chloride-containing chitosan/polyvinylalcohol-based buccal films with unsatisfactory surface pH of 4.89–5.35 [10], suggesting that the newly developed films will not pose any irritation to the buccal mucosal tissues.

Folding endurance values of buccal films dictate their ability to maintain their integrity during handling, transportation, and after buccal application. Satisfactory drug-containing films display folding endurance values of ≥250 as they can withstand repeated folding without breaking [24]. All the studied fluconazole films displayed excellent folding endurance of over 500. The folding endurance values of the optimised sodium alginate/methylcellulose-based fluconazole films were greater than that recorded for hydroxypropyl cellulose/methylcellulose/ethyl cellulose-based felodipine buccal films [15] and HPMC E3/HPMC K4/Carbopol 940-based doxycycline hyclate containing buccal films (>500 versus 212–232 versus > 350) [12]. These findings suggested that the folding endurance of buccal films was dependent on their polymeric constituents and nature of the loaded drug. Moreover, the interaction between sodium alginate and methylcellulose may be greater than those present between cellulose derivatives.

The tensile strength of buccal films quantifies their stretchable nature whereas the resistance of the buccal film formulation to puncture during handling, transportation and buccal application indicates their “elongation at break” (breaking strain) profile. These tensile parameters were assessed to confirm the mechanical profiles of the optimised buccal films. The composition of the buccal films influenced their tensile strength and breaking strain values (Figure 3a,b). For instance, the tensile strength of the fluconazole films increased with an increase in their sodium alginate content (Figure 3a). Film F3 containing 1.6% sodium alginate displayed the highest tensile strength (0.68 ± 0.04 MPa). However, film F3 displayed the lowest breaking strain (12.28 ± 4.51%). Nevertheless, the breaking strain value of F3 was satisfactory. There was no significant statistical difference between the breaking strain of film F2 and film F3 (Figure 3b) containing 1.3% and 1.6% of sodium alginate, respectively (13.62 ± 3.88% versus 12.28 ± 4.51%; *p* > 0.05).

Even though film F1 had the best breaking strain (28.81 ± 1.38%) (Figure 3b), it may be unsatisfactory for buccal application due to its poor tensile strength (0.07 ± 0.01 MPa) (Figure 3a), which will compromise its stretchability, resulting in film puncture and poor patient acceptability. Overall, film F3 exhibited the most promising tensile properties required for the buccal delivery of fluconazole. This finding is in good agreement with those reported for the most promising Eudragit L-100/chitosan-based tenofovir-containing and boronated 4-arm PEG/PVP/MC-based fluconazole-containing vaginal films that exhibited the best tensile strength and least breaking strain [24,34]. In addition, the most promising fluconazole buccal film F3 exhibited improved tensile strength compared to HPMCE3/HPMCK4/Carbopol 934-based doxycycline hyclate-containing buccal films reported by Dinte and coworkers (0.68 ± 0.04 MPa versus 0.39 MPa) [12], suggesting that the type of polymeric constituents of film formulation influenced their mechanical properties.

Interestingly, the most promising previously reported boronated 4-arm PEG/MC/PVP-based fluconazole-containing vaginal films [24] exhibited greater breaking strain value (54%) than the best SA/MC-based fluconazole buccal films in the current study (12%). Nevertheless, both films exhibited comparable satisfactory tensile strength (0.6 MPa versus 0.7 MPa), revealing that the most promising fluconazole-containing buccal films will be suitable for buccal application. This finding may be due to the two film-forming polymeric constituents (methylcellulose and polyvinylpyrrolidone) of the previously reported vaginal film [24] while one film-former (methylcellulose) was employed in the current research to formulate buccal films.

The extent of polymeric film hydration is dependent on the type and physicochemical properties of the constituent polymers [35]. The swelling index data (Table 2) revealed that the sodium alginate content of the film influenced their swelling capabilities. For instance, film F1 containing 1% of sodium alginate exhibited a lesser swelling index than film F3 with sodium alginate content of 1.6% (57 ± 7% versus 70 ± 7%; *p* < 0.05), suggesting that the concentration of sodium alginate in the films influenced their extent of simulant saliva hydration. However, there was no significant statistical difference between the swelling indexes of film F2 and F3 containing 1.3% and 1.6% of sodium alginate, respectively (68 ± 2% versus 70 ± 1%; *p* > 0.05).

Interestingly, the films disintegrated after swelling. This finding could be due to the hydrophilicity of methylcellulose and sodium alginate, inducing water uptake into the polymeric films, facilitating relaxation of stretched, twisted or entangled bioadhesive polymer (sodium alginate), leading to the disentanglement of individual polymer chains, generation of macromolecular network, increased porosity, and the disintegration of the films [36]. Moreover, this disintegration pattern is desirable for the loaded drug to become available at the buccal mucosa surfaces and permeate into the underlying buccal tissues at a controlled rate. Film F3 exhibited the highest disintegration time of 35 min. This finding may be due to the greatest sodium alginate content preventing the dissociation of the film matrix.

The studied SA/MC-based fluconazole films exhibited comparable swelling profile with HPMC/HPC/EC/MC-based felodipine films (57–70% versus 57–80%) [15]. On the other hand, they displayed greater swelling index than Proloc/HPMC/Eudragit RS 100-based rizatriptan benzoate films [13], HPMCE3/HPMCK4/C940-based doxycycline hyclate films [12], chitosan/HPMC or MC or HEC or PVA-based cetylpyridinium chloride films [10] (57–70% versus 20–30% versus 12–43% versus 5–20%). These findings revealed that the nature of the polymeric constituents of the film formulation was a more critical determinant of its swelling potential than the number of polymers present in the films.

Mucoadhesion is a critical parameter that dictates successful buccal drug delivery because insufficient mucoadhesion could displace the buccal film from the site of application, reducing contact time between the drug and the diseased buccal tissues, resulting in therapeutic failure. Cellulose derivatives, such as methylcellulose, and natural polysaccharides, such as sodium alginate, possess hydrogel-forming properties [37,38], which are necessary for mucoadhesion. In addition, sodium alginate could interact with buccal mucosal surfaces via non-covalent bonding between the carboxyl groups of alginate and hydroxyl groups of mucin glycoproteins [38]. Moreover, polymer swelling in simulated saliva facilitate polymer chain uncoiling and improve hydrogen bonding and/or electrostatic interaction between polymer and mucosal surfaces [36].

The sodium alginate content of the fluconazole films dictated their in vitro adhesive property and ex vivo mucoadhesiveness. For instance, the in vitro adhesion time and ex vivo mucoadhesion time values of the formulation F3 containing 1.6% sodium alginate was greater than that of the samples F2 and F1 containing 1.3% and 1.6% of sodium alginate, respectively (5.5 ± 0.3 h; 2.3 ± 0.3 h versus 3.8 ± 0.4 h; 1.6 ± 0.1 h versus 3.0 ± 0.6 h; 1.2 ± 0.3 h). Significant statistical differences between in vitro adhesion time and ex vivo mucoadhesion time values of the studied fluconazole films became evident when the concentration of sodium alginate in the film increased from 1.3% to 1.6% (*p* < 0.05).

This finding is in good agreement with previous studies on boronated 4-arm PEG/PVP/MC-based fluconazole vaginal films, with increased content of the mucoadhesive polymer (boronated 4-arm PEG) in the films resulting in an improved in vitro mucoadhesion time profile of the vaginal films [24]. There was good correlation between the swelling index of fluconazole films and their mucoadhesive profile. For instance, film F3 with the highest in vitro adhesion and ex vivo mucoadhesion time values (5.5 ± 0.3 h; 2.3 ± 0.3 h) displayed the best swelling index (70 ± 1%). The most promising alginate/pectin- based posaconazole-containing buccal films exhibited greater ex vivo porcine buccal mucoadhesion time (3.4 h) than the most promising fluconazole-containing sodium alginate/methylcellulose-based buccal film (2.3 h), which may be due to differences in the equipment used for experimentation. For instance, posaconazole films were evaluated using a modified USP disintegration tester while the fluconazole films were assessed using a shaking water bath technique [11]. Nevertheless, the currently studied fluconazole films exhibited satisfactory ex vivo mucoadhesion time sufficient to facilitate controlled drug release and permeation into the diseased buccal mucosal tissues.

“Mean drug content evaluation” is an important pharmaceutical quality control test for buccal films. USP recommends that drug content of finished drug products should contain 85–115% of active pharmaceutical ingredient [39]. The studied fluconazole films contained satisfactory level of the drug (98.7–113.8%), which suggested that the solvent casting method of preparing the drug-containing films was efficient and the inclusion of appropriate plasticiser (propylene glycol) facilitated uniform drug distribution within the film formulation.

Interestingly, there was a good correlation between the concentration of the mucoadhesive constituent (sodium alginate) of the fluconazole films and their drug content. For instance, the drug content of the fluconazole film F1 containing 1% sodium alginate was lesser than that of the films F2 and F3, containing 1.3% and 1.6% of sodium alginate, respectively (98.7 ± 0.2% versus 99.7 ± 0.2% versus 113.8 ± 0.2%). This could be due to the greater concentration of sodium alginate facilitating retention of greater amount of the drug within the film formulation. This finding is in good agreement with findings obtained for fluconazole-containing vaginal films containing 0.075% of mucoadhesive boronated 4-arm polyethylene glycol (B4PEG) exhibiting greater drug content than the similar film formulation containing 0.05% of B4PEG (107% versus 110%) [24].

The simulated salivary fluid used for the drug release experiment was a mixture of phosphate buffer and methanol, at a volume ratio of 1:1. Methanol was added to the release medium to improve solubilisation of hydrophobic fluconazole in the release medium. The amounts of fluconazole released from films F1, F2 and F3 containing 1%, 1.3% and 1.6% of sodium alginate after 6 h were 4046 ± 109 μg/cm^2^, 4075 ± 359 μg/cm^2^, and 3645 ± 161 μg/cm^2^, respectively. Interestingly, there was no significant statistical differences between the amount of fluconazole released from the studied fluconazole films after 6 h (*p* > 0.05) in simulant salivary fluid (pH 6.8), which might be due to their similar methylcellulose content. This finding is in good agreement with previous studies where two different fluconazole films containing 1.6% methylcellulose and differing boronated 4-arm polyethylene glycol content exhibited similar drug flux (4740–4776 μg/cm^2^) in simulant vaginal fluid (pH 4.2) [24], confirming that the drug release controlling polymer, methylcellulose, was a stronger determinant for fluconazole release profile of the films than the pH of the release medium.

All the studied fluconazole-containing buccal films fitted best with the Higuchi and Korsmeyer–Peppas drug release kinetic model (Table 3). Also, their Korsmeyer–Peppas model-associated diffusional exponent (n) values ranged from 3.27 to 3.63, suggesting that fluconazole was released from the film formulation via simulant salivary fluid diffusion into the fluconazole film and relaxation of the sodium alginate/methylcellulose-based polymeric films [40]. This finding is in good agreement with earlier studies on sodium alginate/polyvinyl alcohol and boronated 4-arm polyethylene glycol/polyvinylpyrrolidone/methylcellulose-based fluconazole films that fitted best with the Higuchi model [24,27], suggesting that the type of polymer and drug used to formulate films influenced their drug release kinetics.

The agar ditch method is an established method for determining the antifungal activity of pharmaceutical semi-solid and film formulations, with the antifungal properties of the drug formulation evaluated in terms of its zone of fungal inhibition (ZOI) [24,41]. Fluconazole solution exhibited superior antifungal activity relative to the studied films F1 to F3 (ZOI: 42.3 mm versus 39.7–40.2 mm) (Table 4). This finding may be due to the lack of polymers in the fluconazole solution which facilitated rapid drug release and antifungal activity, which could result in short-lived therapeutic action in vivo. However, there was no significant statistical differences between the antifungal activity of the fluconazole films F1 to F3 (*p* > 0.05), suggesting that the polymeric constituents of the fluconazole films did not dictate their antifungal activity, and the concentration of fluconazole in the films determined their anticandidal effect.

The anti-*Candida* activity of SA/MC-based fluconazole films was less than that of previously studied Eudragit RS 100/HPMC-based fluconazole oral strips [32] and boronated polyethylene glycol/PVP/MC-based fluconazole vaginal films [24] (ZOI: 39.7–40.2 mm versus 47.0 to 49.7 mm versus 49.2–50.8 mm). These findings may be due to differences in the strain of *Candida* tested as well as the body site where the fungal strains were obtained. Nevertheless, the anti-*Candida* activity for the newly developed fluconazole films were excellent because effective antifungal products exhibit zone of *Candida* inhibition of ≥20 mm [42].

There were no remarkable differences in the organoleptic and mechanical properties of the optimised fluconazole films after storage at 30 ± 2 °C and a relative humidity of 65 ± 5% for 6 months, indicating that the fluconazole buccal films were stable. The sustained stability of the fluconazole films may be due to the favourable interaction between the polymeric constituents and drugs.

## 5. Conclusions and Future Directions

For the first time, mucoadhesive sodium alginate/methylcellulose-based fluconazole buccal films were successfully formulated and characterised for potential treatment of oral candidiasis. FTIR analysis confirmed no significant chemical interactions between fluconazole and the chosen polymers, ensuring drug stability and compatibility with polymeric excipients used to formulate the buccal films. The films exhibited satisfactory organoleptic, physicomechanical, swelling, mucoadhesive properties, drug release profiles, stability, and antifungal activity, indicating their potential for effective treatment of oral candidiasis. Interestingly, all the optimised fluconazole films displayed excellent antifungal activity against *Candida* albicans (zone of *Candida* inhibition: 40 mm). The fluconazole films containing 1.6% SA and 1.6% MC (F3), was the most promising formulation based on its surface pH, tensile strength, swelling index and mucoadhesiveness. It could be used as an alternative dosage form to the marketed fluconazole gel for the potential treatment of oral candidiasis. The new SA/MC-based buccal films could result in drug dosage reduction from 150 mg to 5 mg as well as avoidance of side effects associated with oral and systemic drug delivery and improved patient compliance.

Future research could explore a combination of boronated polymer and sodium alginate as the mucoadhesive polymer and the influence of the type and concentration of polymers on the mucoadhesive and antifungal properties of the novel film formulation will be investigated. In addition, taste of buccal films would be evaluated as this organoleptic attribute could impact patient acceptability of the dosage form.

## Figures and Tables

**Table 1:** Composition of fluconazole-containing buccal film formulations.

Films	SA (%)	MC (%)	GLY (%)	PG (%)	FLU (mg/cm^2^)
AF1	1	1.8	10	-	5
BF1	1	1.6	10	-	5
F1	1	1.6	-	10	5
AF2	1.3	1.8	10	-	5
BF2	1.3	1.6	10	-	5
F2	1.3	1.6	-	10	5
AF3	1.6	1.8	10	-	5
BF3	1.6	1.6	10	-	5
F3	1.6	1.6	-	10	5

**Key:** SA: sodium alginate; MC: methylcellulose; GLY: glycerol; PG: propylene glycol; FLU: fluconazole; AF1, AF2, AF3 (viscous film-forming solutions), BF1, BF2, BF3 (sticky, unstable films); F1 = SA1%/MC1.6%, F2 = SA1.3%/MC1.6%, F3 = SA1.6%/MC1.6% (optimised films).

**Table 2:** Physicomechanical properties of fluconazole film formulations.

Samples	Weight (g)	Thickness (mm)	Folding Endurance	Surface pH	Disintegration Time in SSF (min)	Swelling Index (%)
F1	0.10 ± 0.01	0.09 ± 0.01	>500	7.61 ± 0.03	5	57 ± 7
F2	0.11 ± 0.01	0.08 ± 0.01	>500	7.18 ± 0.03	10	68 ± 2
F3	0.12 ± 0.01	0.09 ± 0.01	>500	7.13 ± 0.05	35	70 ± 1

**Key:** F1: Fluconazole/1% SA/1.6% MC; F2: Fluconazole/1.3% SA/1.6% MC; F3: Fluconazole/1.6% SA/1.6% MC; SSF: simulated salivary fluid.

**Table 3:** Drug release data for fluconazole films fitted to various drug kinetic models—zero-order, first-order, Higuchi and Korsmeyer–Peppas models.

Kinetic Models	Zero		First		Higuchi		Korsmeyer–Peppas		
Sample	r2	k1	r2	k2	r2	k3	r2	k4	n-Value
F1	0.928	9.8193	0.6674	1.0597	0.9796	−28.295	0.9722	1.4492	3.3368
F2	0.8986	20.211	0.7167	1.333	0.9689	−10.707	0.981	1.5762	3.6292
F3	0.8897	12.026	0.668	1.095	0.9639	−14.211	0.9633	1.4216	3.2732

Key: F1: Fluconazole/1% SA/1.6% MC; F2: Fluconazole/1.3% SA/1.6% MC; F3: Fluconazole/1.6% SA/1.6% MC.

**Table 4:** Zones of inhibition (mm) against *C. albicans* for fluconazole solution and films; data presented as mean ± SD; n = 3.

Sample	Mean ± SD
Fluconazole solution	42.3 ± 0.5
F1	39.7 ± 0.5
F2	40.2 ± 0.2
F3	39.7 ± 0.5

Key: F1: Fluconazole/1% SA/1.6% MC; F2: Fluconazole/1.3% SA/1.6% MC; F3: Fluconazole/1.6% SA/1.6% MC.

## Data Availability

Dataset available on request from the authors.

## Supplementary Materials

The following supporting information can be downloaded at https://www.mdpi.com/article/10.3390/pharmaceutics18060748/s1, Table S1: Chromatographic (UV) Analytical Method Validation parameters for fluconazole quantification in simulated salivary fluid/methanol mixture (1:1; pH 6.8), with accuracy and precision of the UV method determined from three graded fluconazole concentrations (low, medium and high), i.e., 5 μg/mL, 25 μg/mL and 75 μg/mL; n = 3; Figure S1: Fluconazole Calibration Curve prepared using standard solutions of fluconazole in phosphate buffer/methanol (1:1), pH 6.8; n = 3; Figure S2: Images revealing zones of *C. albicans* inhibition exhibited by fluconazole solution and fluconazole films F1 to F3.

## References

[1] Singh A., Verma R., Murari A., Aqrawal A. (2014). Oral candidiasis: An overview. J. Oral Maxillofac. Pathol..

[2] Ashman R.B., Farah C.S., Fidel P.L., Huffnagle G.B. (2005). Oral candidiasis: Clinical manifestations and cellular adaptive host responses. Fungal Immunology: From an Organ Perspective.

[3] Vila T., Sultan A.S., Montelongo-Jauregui D., Jabra-Rizk M.A. (2020). Oral Candidiasis: A Disease of Opportunity. J. Fungi.

[4] Mushi M.F., Bader O., Taverne-Ghadwal L., Bii C., Groß U., Mshana S.E. (2017). Oral candidiasis among African human immunodeficiency virus-infected individuals: 10 years of systematic review and meta-analysis from sub-Saharan Africa. J. Oral Microbiol..

[5] Ambe N.F., Longdoh N.A., Tebid P., Bobga T.P., Nkfusai C.N., Ngwa S.B., Nsai F.S., Cumber S.N. (2020). The prevalence, risk factors and antifungal sensitivity pattern of oral candidiasis in HIV/AIDS patients in Kumba District Hospital, Southwest Region, Cameroon. Pan Afr. Med. J..

[6] Taylor M., Brizuela M., Raja A. (2023). Oral Candidiasis.

[7] Pasko M.T., Piscitelli S.C., Van Slooten A.D. (1990). Fluconazole: A new triazole antifungal agent. DICP.

[8] Ashok A., Mangalore R.P., Morrissey C.O. (2022). Azole therapeutic drug monitoring and its use in the management of invasive fungal disease. Curr. Fungal Infect. Rep..

[9] Shipp L., Liu F., Kerai-Varsani L., Okwuosa T.C. (2022). Buccal films: A review of therapeutic opportunities, formulations & relevant evaluation approaches. J. Control. Release.

[10] Abouhussein D., el Nabarawi M.A., Shalaby S.H., El-Bary A.A. (2020). Cetylpyridinium chloride chitosan blended mucoadhesive buccal films for treatment of pediatric oral diseases. J. Drug Deliv. Sci. Technol..

[11] Szekalska M., Czajkowska-Kosnik A., Maciejewska B., Misztalewska-Turkowicz I., Wilczewska A.Z., Bernatoniene J., Winnicka K. (2023). Mucoadhesive Alginate/pectin films crosslinked by calcium carbonate as carriers of a model antifungal drug—Posaconazole. Pharmaceutics.

[12] Dinte E., Muntean D.M., Andrei V., Bosca B.A., Dudescu C.M., Barbu-Tudoran L., Borodi G., Andrei S., Gal A.F., Rus V. (2023). In Vitro and In Vivo Characterisation of a Mucoadhesive Buccal Film Loaded with Doxycycline Hyclate for Topical Application in Periodontitis. Pharmaceutics.

[13] Nair A.B., Shah J., Jacob S., Al-Dhubiab B.E., Patel V., Sreeharsha N., Shinu P. (2021). Development of mucoadhesive buccal film for rizatriptan: In Vitro and In Vivo evaluation. Pharmaceutics.

[14] Ammar H.O., Ghorab M.M., Mahmoud A.A., Shahin H.I. (2017). Design and in vitro/in vivo evaluation of ultra-thin mucoadhesive buccal film containing fluticasone propionate. AAPS PharmSciTech.

[15] Haritha K., Devi N., Durga A., Himaja V., Mounika P., Vyshnavi V. (2018). Buccal Films Containing Felodipine: In Vitro and In Vivo Evaluation. Int. J. Pharm. Sci. Rev. Res..

[16] Soradech S., Williams A., Khutoryanskiy V. (2025). Synthesis of poly (2-hydroxyethyl ethyleneimine) and its mucoadhesive film formulations when blended with chitosan for buccal delivery of haloperidol. Macromol. Biosci..

[17] Abdella S., Afinjuomo F., Song Y., Upton R., Garg S. (2022). Mucoadhesive Buccal Film of Estradiol for Hormonal Replacement Therapy: Development and In-Vivo Performance Prediction. Pharmaceutics.

[18] Bastos F., Pinto A.C., Nunes A., Simoes S. (2022). Oromucosal products–Market landscape and innovative technologies: A review. J. Control. Release.

[19] Srivastava N., Aslam S. (2022). Recent advancements and patents on buccal drug delivery systems: A comprehensive review. Recent Pat. Nanotechnol..

[20] Jacob S., Nair A.B., Boddu S.H., Gorain B., Sreeharsha N., Shah J. (2021). An updated overview of the emerging role of patch and film-based buccal delivery systems. Pharmaceutics.

[21] Drug Bank (2023). Fluconazole. https://go.drugbank.com/drugs/DB00196.

[22] Costa M.J., Marques A.M., Pastrana L.M., Teixeira J.A., Sillankorva S.M., Cerqueira M.A. (2018). Physicochemical properties of alginate-based films: Effect of ionic crosslinking and mannuronic and guluronic acid ratio. Food Hydrocoll..

[23] Khan S., Boateng J.S., Mitchell J., Trivedi V. (2015). Formulation, characterisation and stabilisation of buccal films for paediatric drug delivery of omeprazole. AAPS PharmSciTech.

[24] Kolawole O.M., Okeke P.K. (2025). Formulation and Evaluation of boronated 4-arm polyethylene glycol/polyvinyl pyrrolidone/methylcellulose-based fluconazole films for the potential treatment of vaginal candidiasis. J. Drug Deliv. Sci. Technol..

[25] Pamlenyi K., Kristo K., Jojart-Lacz K., Regdon G. (2021). Formulation and optimization of sodium alginate polymer film as a Buccal Mucoadhesive Drug Delivery System Containing Cetirizine Dihydrochloride. Pharmaceutics.

[26] U.S. Food & Drug Administration Code for Federal Regulations Title 21 Part 184—Direct Food Substances Affirmed as Generally Recognized as Safe. https://www.accessdata.fda.gov/scripts/cdrh/cfdocs/cfcfr/CFRSearch.cfm?fr=184.1724.

[27] Patel S.K., Shah D.R., Tiwari S. (2015). Bioadhesive films containing fluconazole for mucocutaneous candidiasis. Indian J. Pharm. Sci..

[28] ICH (2003). ICH Harmonized Tripartite Guideline—Stability Testing of New Drug Substances and Products Q1A (R2). https://database.ich.org/sites/default/files/Q1A%28R2%29%20Guideline.pdf.

[29] Govindarajan A., Bistas K.G., Ingold C.J., Patel P., Aboeed A. Fluconazole. StatPearls.

[30] Moura-Alves M., Souza V.G.L., Silva J.A., Esteves A., Pastrana L.M., Saraiva C., Cerqueira M.A. (2023). Characterisation of Sodium Alginate-Based Films Blended with Olive Leaf and Laurel Leaf Extracts Obtained by Ultrasound-Assisted Technology. Food.

[31] Hashemi M., Ramezani V., Seyedabadi M., Ranjbar A.M., Jafari H., Honarvar M., Fanaei H. (2017). Formulation and Optimization of oral. mucoadhesive patches of Myrtus Communis by Box Behnken Design. Adv. Pharm. Bull..

[32] Rençber S., Karavana S.Y., Yilmaz F.F., Eraç B., Nenni M., Gurer-Orhan H., Limoncu M.H., Güneri P., Ertan G. (2019). Formulation and evaluation of fluconazole loaded oral strips for local treatment of oral candidiasis. J. Drug Deliv. Sci. Technol..

[33] Sandri G., Rossi S., Ferrari F., Bonferoni M.C., Muzzarelli C., Caramella C. (2004). Assessment of chitosan derivatives as buccal and vaginal penetration enhancers. Eur. J. Pharm. Sci..

[34] Martín-Illana A., Cazorla-Luna R., Notario-Pérez F., Rubio J., Ruiz-Caro R., Tamayo A., Veiga M.D. (2022). Eudragit^®^ L100/chitosan composite thin bilayer films for intravaginal pH-responsive release of Tenofovir. Int. J. Pharm..

[35] Avachat A.M., Gujar K.N., Wagh K.V. (2013). Development and evaluation of tamarind seed xyloglucan-based mucoadhesive buccal films of rizatriptan benzoate. Carbohydr. Polym..

[36] Boateng J., Okeke O. (2019). Evaluation of Clay-Functionalized Wafers and Films for Nicotine Replacement Therapy via Buccal Mucosa. Pharmaceutics.

[37] Conti S., Maggi L., Segale L., Machiste E.O., Conte U., Grenier P., Vergnault G. (2007). Matrices containing NaCMC and HPMC: 2. Swelling and release mechanism study. Int. J. Pharm..

[38] Szekalska M., Puciłowska A., Szymańska E., Ciosek P., Winnicka K. (2016). Alginate: Current Use and Future Perspectives in Pharmaceutical and Biomedical Applications. Int. J. Polym. Sci..

[39] USP/NF (2020). Uniformity of Dosage Units. United States Pharmacopeia and National Formulary. USP 43-NF 38: General Chapter <905> Uniformity of Dosage Units, United States Pharmacopeia Convention. https://www.usp.org/sites/default/files/usp/document/harmonization/excipients/m99694.pdf.

[40] Baggi R.B., Kilaru N.B. (2016). Calculation of predominant drug release mechanism using Peppas-Sahlin model, Part-1 (Substitution method): A linear regression approach. Asian J. Pharm. Technol..

[41] Ogedengbe O.T., Kolawole O.M. (2024). Formulation and evaluation of fluconazole emulgels for potential treatment of vaginal candidiasis. Heliyon.

[42] Nurcahyanti A.D.R., Liliana M., Surja S.S. (2024). Inhibitory activity of Lagerstreomia speciosa extract against Candida albicans, Aspergillus fumigatus, and Aspergillus flavus. J. Appl. Pharm. Sci..

