# Supplementary material for: Formulation and Evaluation of Fluconazole Containing Sodium Alginate/Methylcellulose-Based Buccal Films for Potential Treatment of Oral Candidiasis
Source: Pharmaceutics. 2026 Jun 18;18(6):748. doi: 10.3390/pharmaceutics18060748 (PMC13307397; doi:10.3390/pharmaceutics18060748)
Supplement: Supplementary file 1 [file pharmaceutics-18-00748-s001.zip › pharmaceutics-4350065-supplementary.pdf]

Supplementary Information

Formulation and Evaluation of Fluconazole Containing Sodium Alginate/Methylcellulose-Based Buccal Films for Potential Treatment of Oral Candidiasis

Adekunle Oduneye Odularu [1], Anuoluwapo Temitope Adesegun [1], Chukwuemeka Paul Azubuike [1] and Oluwadamilola Miriam Kolawole [1,2,*]

[1] Department of Pharmaceutics and Pharmaceutical Technology, University of Lagos, Lagos +23401, Nigeria; odular1@yahoo.com (A.O.O.); desegunanu@gmail.com (A.T.A.); cazubuike@unilag.edu.ng (C.P.A.)
[2] School of Pharmacy, De Montfort University, The Gateway, Leicester LE1 9BH, UK

* Correspondence: oluwadamilola.kolawole@dmu.ac.uk

Table S1: Chromatographic (UV) Analytical Method Validation Parameters for fluconazole quantification in simulant salivary fluid/methanol mixture (1:1; pH 6.8), with accuracy and precision of the UV method determined from three graded fluconazole concentrations (low, medium, and high), i.e. 5 µg/mL, 25 µg/mL, and 75 µg/mL, n = 3

Nominal Concentrations (µg/mL)	LLOQ (5.0)	MLOQ (25.0)	HLOQ (75.0)	WHO Limit of specification
Experimental Values (µg/mL)	5.50<br>5.52<br>5.30	24.80<br>24.03<br>24.18	74.09<br>74.04<br>74.18	
Mean Experimental Value (µg/mL)	5.44	24.34	74.10	
Standard Deviation (SD; ±)	0.12	0.41	0.07	
Relative Standard Deviation (RSD) %	1.87	1.24	0.07	≤ 15
Mean Accuracy (%)	98.1	98.8	99.9	≥ 85
Mean deviation (%)	1.71	1.22	0.07	≤ 15
Coefficient of Variation (CV) %	2.21	1.68	0.09	≤ 15
Variance	0.01	0.17	0.01	
Correlation Coefficient (R^2)		0.9988		≥ 0.90
Limit of detection (fluconazole)		1.2 µg/mL		
Limit of quantification (fluconazole)		4 µg/mL		

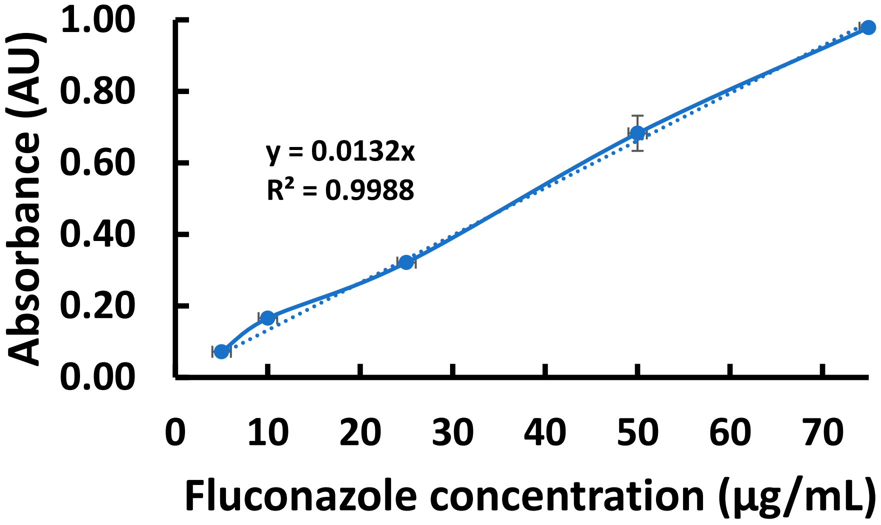

Figure S1: Fluconazole Calibration Curve prepared using standard solutions of fluconazole in phosphate buffer/methanol (1:1), pH 6.8; n=3

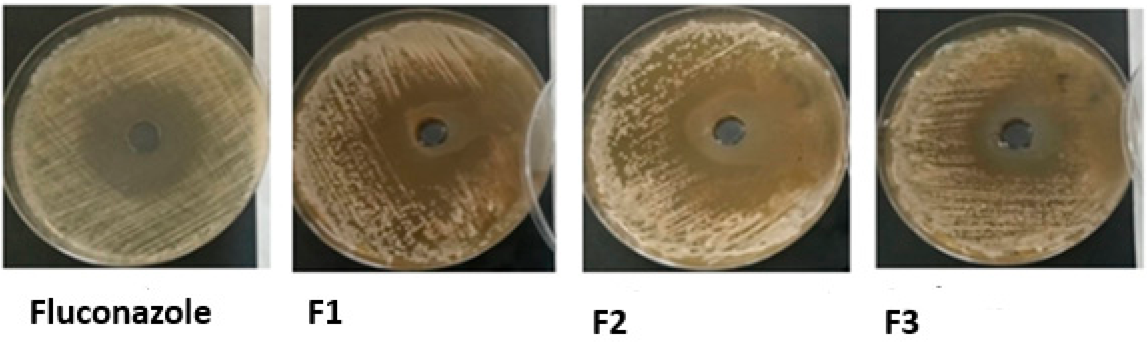

Figure S2: Images revealing zones of *C. albicans* inhibition exhibited by fluconazole solution and fluconazole films F1 to F3; F1=SA1%/MC1.6%; F2= SA1.3%/MC1.6%, F3 = SA1.6%/MC1.6 % (n = 3).
